# Activation of the NFκB signaling pathway in IL6+CSF3+ vascular endothelial cells promotes the formation of keloids

**DOI:** 10.3389/fbioe.2022.917726

**Published:** 2022-08-23

**Authors:** Delin Liu, Yidi Zhang, Lisha Zhen, Rong Xu, Zhenling Ji, Zheng Ye

**Affiliations:** ^1^ Department of General Surgery, Institute for Minimally Invasive Surgery, Affiliated Zhongda Hospital, Medical School, Southeast University, Nanjing, China; ^2^ Department of Endcrinology, Affiliated Zhongda Hospital, Medical School, Southeast University, Nanjing, China; ^3^ School of Statistics, Renmin University of China, Beijing, China; ^4^ Beijing Sankuai Online Technology Co.,Ltd, Dhaka, Bangladesh

**Keywords:** keloid, IL6, endothelial cells (ECs), scRNAseq, NFkB (RelA)

## Abstract

**Background:** Keloid is a disease caused by abnormal proliferation of skin fibres, the causative mechanism of which remains unclear.

**Method:** In this study, endothelial cells of keloids were studied using scRNAseq combined with bulk-RNAseq data from keloids. The master regulators driving keloid development were identified by transcription factor enrichment analysis. The pattern of changes in vascular endothelial cells during keloid development was explored by inferring endothelial cell differentiation trajectories. Deconvolution of bulkRNAseq by CIBERSORTX verified the pattern of keloidogenesis. Immunohistochemistry for verification of the lesion process in keloid endothelial cells.

**Results:** The endothelial cells of keloids consist of four main cell populations (MMP1+ Endo0, FOS + JUN + Endo1, IL6+CSF3+Endo2, CXCL12 + Endo3). Endo3 is an endothelial progenitor cell, Endo1 is an endothelial cell in the resting state, Endo2 is an endothelial cell in the activated state and Endo0 is an endothelial cell in the terminally differentiated state. Activation of the NFΚB signaling pathway is a typical feature of Endo2 and represents the early skin state of keloids.

**Conclusion:** We have identified patterns of vascular endothelial cell lesions during keloidogenesis and development, and have found that activation of the NFΚB signaling pathway is an essential feature of keloid formation. These findings are expected to contribute to the understanding of the pathogenesis of keloids and to the development of new targeted therapeutic agents for the lesional characteristics of vascular endothelial cells.

## Background

Keloid is a disease caused by abnormal proliferation of skin fibres during the prognosis of a wound, which is mainly characterised by excessive deposits of collagen secreted by fibroblasts (Babu et al., 1989; Luo et al., 2001). Keloid scars are often accompanied by pain and itching and can significantly affect the quality of life of the patients. Although keloids do not pose an immediate threat to a patients’ life, they can cause disruption to the patients’ appearance and impairment of physical activity, causing them great physical and emotional pain (Reinholz et al., 2015; Aluko-Olokun et al., 2018). The molecular mechanisms underlying the formation and progression of keloids are complex. Due to the lack of understanding of the pathogenesis of keloids, current treatments for keloids are limited and have a high recurrence rate (Gupta and TJTJoL, 2011; Park and Chang, 2012). The available evidence suggests that keloid formation is the result of an imbalance between increased collagen and ECM synthesis and decreased degradation of these products (Sidgwick and Bayat, 2012; Macarak et al., 2021). Overexpression of inflammatory factors overactivates keloid fibroblasts resulting in an increase in ECM collagen synthesis (Messadi et al., 2004). Vascular endothelial cells play an important role in the development and progression of keloids. Several studies have shown that abnormal vascular endothelial cell function and their interaction with immune cells and fibroblasts can influence the deposition of extracellular matrix and the progression of inflammation during keloid formation (Ehrlich et al., 1994; Matsumoto et al., 2017). RNA sequencing technology (RNA-seq) allows the transcriptional levels of genes in mixed cell populations to be assessed. It is currently being used extensively in the field of keloid research (Onoufriadis et al., 2018; Matsumoto et al., 2020). Single-cell RNA sequencing (RNA-seq) allows the transcriptome level of a large number of cells to be studied at single-cell resolution and has been applied to the study of the mechanisms of keloid formation (Xie et al., 2021a; Ascensión et al., 2022). A recent study used the single cell transcriptome to identify four main subpopulations of keloid fibroblasts, secretory papillae, secretory reticulocytes, mesenchymal cells and pro-inflammatory cells. Of these fibroblasts, mesenchymal fibroblasts are critical for collagen overexpression in keloids (Deng et al., 2021). However, there are fewer studies exploring the characteristics of endothelial cells in keloids at the single cell level. In this study, we used scRNA-seq combined with bulk-RNA-seq to comparatively analyse the transcriptome characteristics of endothelial cells between keloid skin tissue and normal keloid. Our study provides insight into the role played by endothelial cells in the development of keloid scars, reveals the pathogenesis of keloid scars from endothelial cell changes, and provides potential targets for drug therapy.

## Materials and method

### Data collection

Single-cell transcriptome sequencing data [GSE163973 (16)] of keloid tissues and surrounding tissues from three individuals were obtained from the GEO database. In addition 64 skin samples from GSE113621 (13) (13 general population scars day 0, 14 general population scars day 42, 19 keloid susceptible population day 0, 18 keloid susceptible population day 42) were obtained from the GEO database; 11 keloid endothelial cell samples from GSE121618 (12).

### Single cell data processing flow

Seurat 4.0.1 (17) was used for the analysis process of single cell data. For separate analysis of endothelial cells, we first obtained processed Rdata from GSE163973, integrated the six samples with harmony, and then clustered the single cells using RunUMAP, FindNeighbors, and FindClusters (resolution = 0.8). In the clustered UMAP 2D scatterplot, endothelial cells were selected and the data were integrated in a separate harmony process. By adjusting the resolution = 0.2, we obtained 4 clusters of endothelial cells for subsequent comparison.

### Transcription factor activity analysis

The R package dorothea [(Butler et al., 2018); (Garcia-Alonso et al., 2019)] was used for transcription factor regulation analysis for scRNAseq and bulk-RNAseq. The transcription factor regulatory networks with higher confidence (A,B,C) were selected in Dorothea_hs for subsequent analysis. Dorothea first constructed a regulatory network of transcription factor signatures based on GTEx [(GTEx Consortium, 2015); (Gulati et al., 2020); (Rusk, 2019)] data, public ChIP-seq data and the literature, and then used viper to infer the activity of transcription factors.

### Analysis of cell differentiation trajectories

CytoTRACE (20) is a computational framework for reconstructing the relative differentiation status of single-cell RNA sequencing data based on the expression profile of genes. The differentiation status (Wu et al., 2021) of cells in scRNA-seq data can be inferred without relying on any *a priori* information. The differentiation status of endothelial cells was analysed using CytoTRACE. A KNN graph containing information on the undirected links between cells was first constructed. Then, CytoTRACE was used to calculate the proposed time ordering of cells. Finally, a transfer matrix was constructed based on the KNN graph and the proposed time, and projected onto a UMAP scatter plot.

### Inference of cellular composition

The CIBERSORTX (21) computational framework allows the scoring of components of a cell population to be obtained based on the deconvolution of gene expression profile data from a mixed cell population. Firstly, a feature matrix of endothelial cells was constructed using endothelial cells from the GSE163973 dataset. Then, the feature matrix was used to deconvolute GSE113621 and GSE121618 respectively, and the absolute scores of the four endothelial cells (Endo0, Endo2, Endo3, and Endo4) in the sample were finally obtained.

### Immunohistochemistry

Pathological sections included four patients with keloid and Para-keloid tissues were taken from each patient for immunohistochemical staining. The studies involving human participants were reviewed and approved by IEC for Clinical Research of Zhongda Hospital, Affilliated to Southeast University. The patients provided written informed consent to participate in this study. The monoclonal antibody mouse anti-human RELA was purchased from Abway (1:200 dilution) and the chromogenic kit was purchased from DAKO (K5007, 1:100 dilution). PBS buffer (Na_2_HPO_4_ 8.1 mmol/L, KH_2_PO_4_ 4.5 mmol/L, NaCl 137 mmol/L, 2.7 mmol/L, pH 7.4) was shaken and washed. The sections were then sealed by dehydration in the conventional way.

### Immunofluorescence

The pathological tissue sections were obtained from Zhongda Hospital, which had passed the ethical review and obtained informed consent. Immerse three Keloid slices in xylene 1 (15 min), xylene 2 (15 min), absolute ethanol 1 (5 min), absolute ethanol 2 (5 min), 95% ethanol (5 min), 85% ethanol (5 min), 75% ethanol (5 min), and finally rinse the slides with water. Antigen retrieval usually uses 1× citric acid (PH6.0) as the repair solution, repairs with medium-high fire for 5 min, and cools to room temperature. Prepare 3% H2O2 with water, put the slices in the solution, and incubate at room temperature for 20 min. Draw circles around the tissue with a histochemical pen, and then drip PBST on the tissue to wet the tissue. Add tunel reagent dropwise to the tissue, incubate at 37°C for 60 min, and wash the slides three times with PBST for 5 min each time. Block with 10% goat serum and incubate at 37°C for 30 min. Dilute the primary antibody (CD31, Abcam, No. Ab182981) with antibody diluent according to a certain concentration (1/2000), incubate overnight at 4°C, or incubate at 37°C for 2 h. Secondary antibodies were prepared with PBST and incubated at 37°C for 1 h. TSA-570 was added dropwise to the tissue, incubated at 37°C for 30 min, and then washed with PBS for 3 times, 5 min each time. The slides were placed in 1× citric acid, placed in a microwave oven for 5 min on high heat, and cooled to room temperature. Block with 10% goat serum and incubate at 37°C for 30 min. Dilute (1/500) the primary antibody (RELA, CST, No. 8284) with antibody diluent according to a certain concentration, incubate at 4°C overnight, or incubate at 37°C for 2 h. Secondary antibodies were prepared with PBST and incubated at 37°C for 1 h. TSA-670 was added dropwise to the tissue, incubated at 37°C for 30 min, and then washed with PBS for 3 times, 5 min each time. DAPI was added dropwise to the tissue, incubated at room temperature for 5 min, and then washed with PBS for 3 times, 5 min each time. The liquid was shaken dry, anti-fluorescence mounting medium was added dropwise to the tissue, and a coverslip was placed. Store the prepared fluorescent film at 4°C in the dark. All TSA reagents were prepared in PBST containing 0.003% H2O2, and the dilution ratio was 1/1,000.

### Statistics

R 4.0.1 is used for statistical analysis and plotting. The R package ggplot2 is used to plot and embellish statistical images. The R package ggpubr was used for mean testing of grouped variables (student-*t* test). The R package clusterproflieR (22) was used for functional enrichment analysis of genes (KEGG, GO). The STRING database was used for the protein-protein interaction network and cytoscape 3.8.1 was used to visualize the network. *p*< 0.05 was considered to be statistically significant.

## Results

### The endothelial cells of keloid are divided into four subgroups

**FIGURE 1:**
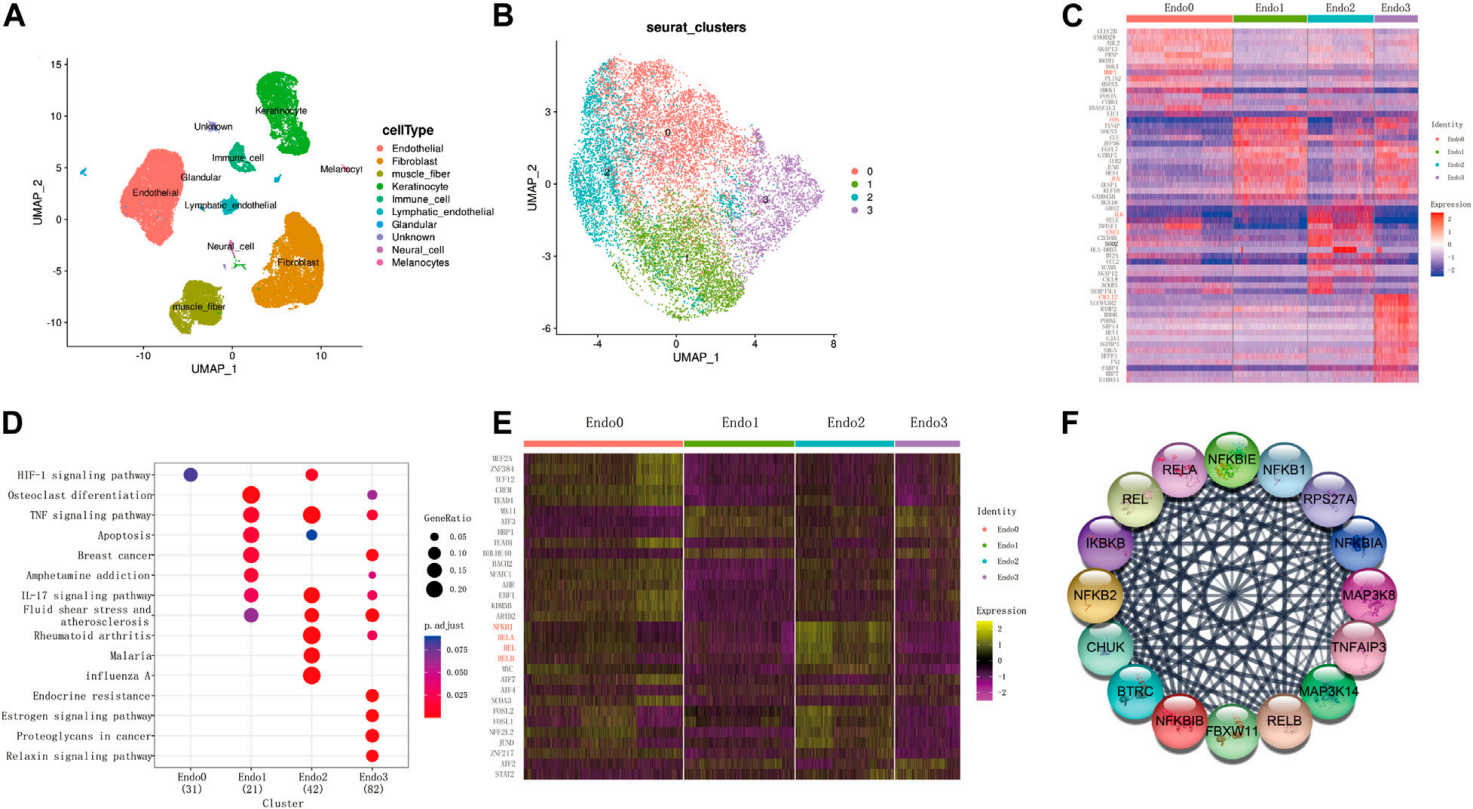
Single-cell transcriptome identifies four subpopulations of keloidal vascular endothelial cells. **(A)** UMAP two-dimensional scatter plot of the GSE163973 keloid single cell dataset after dimensional reduction and clustering. **(B)** Endothelial cells can be divided into four subpopulations. **(C)** Top20 differentially expressed genes for the four endothelial cell subpopulations (wilcoxon-test, log2FC > 0.05, adj. *p* < 0.05). **(D)** Functional enrichment analysis of highly expressed genes from each subpopulation. **(E)** Transcription factor enrichment analysis (wilcoxon-test, log2FC > 0.25, adj. *p* < 0.05). **(F)** Transcription factor Protein-Protein-Interaction network of Endo2.

To explore the regulatory changes in endothelial cells during keloid formation, we obtained transcriptomic data from 40,655 keloid and paraceloid cells using the GSE163973 dataset. The Uniform Manifold Approximation and Projection (UMAP) clustering results show that these cells can be correctly classified into 10 cell types (Figure 1A). We found that endothelial cells consisted of four main cell populations (Endo0, Endo1, Endo2, and Endo3, Figure 1B) by further downscaling clustering of endothelial cells. Genes with differential expression changes in the four endothelial cells were obtained by differential expression analysis (*t*.test, |log2FC|>0.25, p. adj<0.01), and we defined Endo0 as MMP1+ Endothelial cells, Endo1 as FOS + JUN + Endothelial cells, Endo2 as IL6+CSF3+ Endothelial cells, and Endo3 is defined as CXCL12 + Endothelial cells (Figure 1C). Through functional enrichment analysis of highly expressed genes in each of the four states of endothelial cells (KEGG database), it was found that genes upregulated in Endo0 were mainly involved in the HIF-1 signaling pathway; genes upregulated in Endo1 were mainly involved in Osteoclast differentiation, TNF signaling pathway, Apoptosis; Endo2 upregulated genes were mainly involved in IL-17 signaling pathway, Fluid shear stress and atherosclerosis, Rheumatoid arthritis; Endo3 upregulated genes were mainly involved in Endocrine resistance, Estrogen signaling pathway, Proteoglycans in cancer and other signaling pathways (Figure 1D). The transcription factor activities of the four endothelial cells were assessed by dorothea. The results showed that the transcription factors activated by Endo0 were mainly MEF2A, TCF12, KDM5B, and NFATC1 etc.; the transcription factors activated by Endo1 were mainly MXI1, ATF3, and HBP1 etc.; the transcription factors activated by Endo2 were mainly NFΚB1, RELA, REL, and RELB etc.; the transcription factors activated by Endo3 were mainly ATF2, STAT2, etc. (Figure 1E). We constructed a PPI regulatory network for the transcription factors of Endo2 and found that these transcription factors were mainly involved in the NFΚB signaling pathway (Figure 1F).

### Endothelial cell differentiation during keloid formation

**FIGURE 2:**
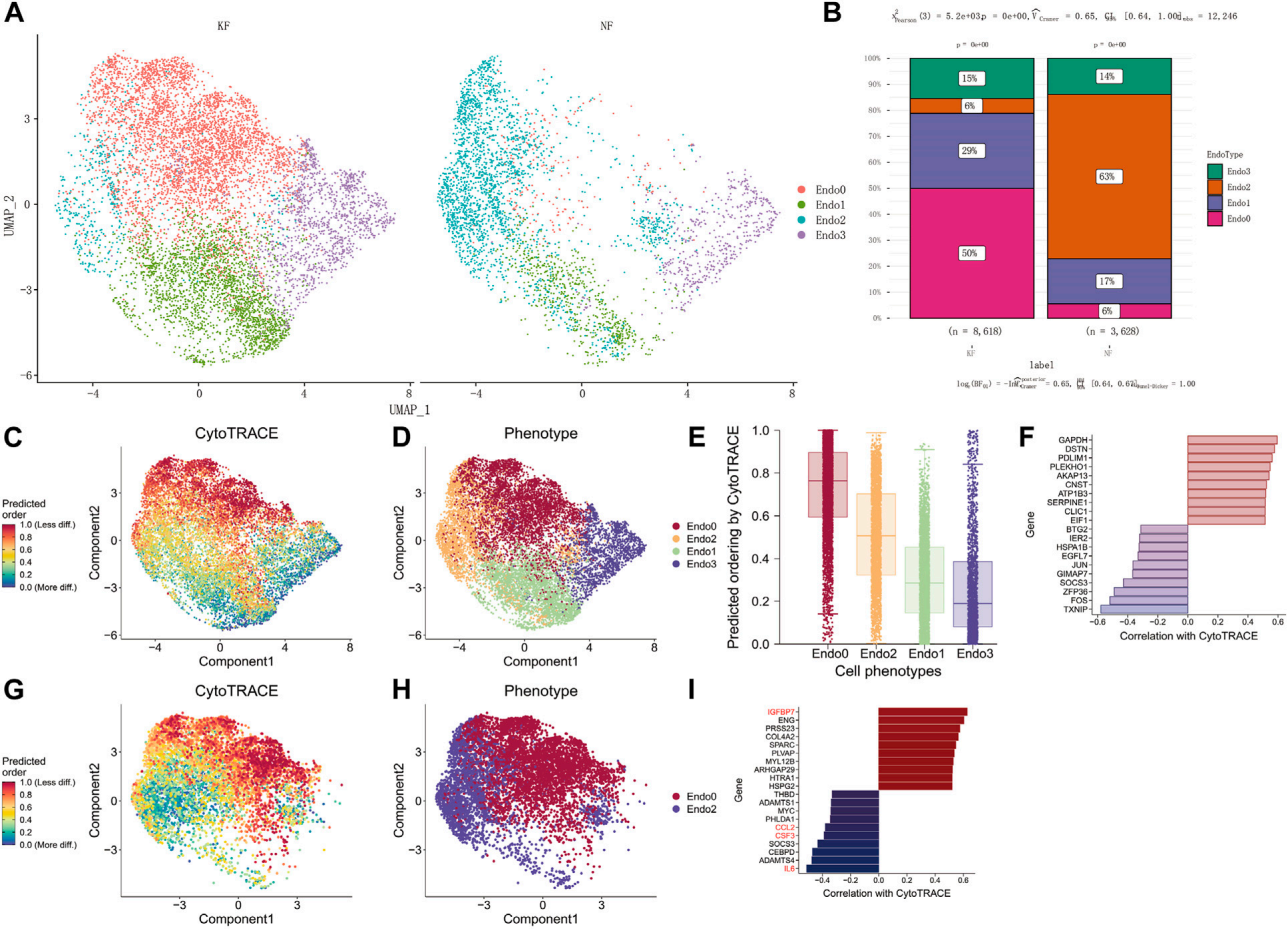
Endo2 differentiation to Endo0 is a major feature of keloidogenesis and development. **(A)** Differential cellular distribution of endothelial cells in NF and KF in the GSE163973 single-cell sequencing dataset. **(B)** Comparison of four subpopulations of NF and KF endothelial cells with a significant difference in the distribution of endothelial cells between the two (chi-square test, *p* < 0.001). **(C–E)** Differentiation trajectory of keloid endothelial cells. The lowest differentiation of Endo3 and the highest differentiation of Endo0 can be observed from the graph. **(F)** The major genes associated with endothelial cell differentiation. **(G–I)** Genes involved in the differentiation from Endo2 to Endo0. Genes including IL6, ADAMTS4, CEBPD, SOCS3, CSF3, and CCL2 inhibit endothelial cell differentiation, while genes such as IGFBP7, ENG, PRSS23, and COL4A2SPARC promote endothelial cell differentiation.

Next, we compared the differences in the proportion of the four types of endothelial cells in keloid and keloid-adjacent tissue (Figure 2A). The results showed that the proportion of Endo0 cells in keloid tissue was 50%, compared to 6% in para-keloid tissues. In contrast, the proportion of Endo2 in keloid tissues was 6%, compared to 63% in para-keloid tissues. Endo1 was equally high in keloids (keloids vs. para-keloid 29% vs. 17%). Endo3, on the other hand, was not very different (keloids vs. para-keloid 15% vs. 17%) (Figure 2B). This result illustrates that reduced Endo2 and elevated Endo0 are the main pathological processes in keloid scars. To further elucidate the characteristics of endothelial cell changes during keloidogenesis, Cytotrace was applied to infer the degree of differentiation of the four endothelial cell types. The results show that Endo3- > Endo1- > Endo2- > and Endo0 is the main process of keloid differentiation (Figures 2C–E). In addition, we found that TXNIP, FOS, ZFP36, SOCS3, GIMAP7, and JUN were the main genes that prevented endothelial cell differentiation; while GAPDH, DSTN, PDLIM1, PLEKHO, AKAP13, CNST, ATP1B3, SERPINE1, CLIC1, and EIF1 were the main genes that drove the differentiation of endothelial cells towards the terminal endo0 (Figure 2F). In conjunction with the previous analysis, we suggest that there is an important relationship between the differentiation of Endo2 to Endo0 and the development of keloids. Therefore, the process of differentiation from Endo2 to Endo0 was further explored. The results showed that IL6, ADAMTS4, CEBPD, SOCS3, CSF3, and CCL2 were the main regulatory genes for maintaining endothelial cell status, while IGFBP7, ENG, PRSS23, COL4A2, and SPARC were the main regulatory genes for promoting endothelial cell differentiation (Figures 2G–I).

### Bulk-RNAseq verifies that differentiation of Endo2 to Endo0 is an important cause of keloid formation

**FIGURE 3:**
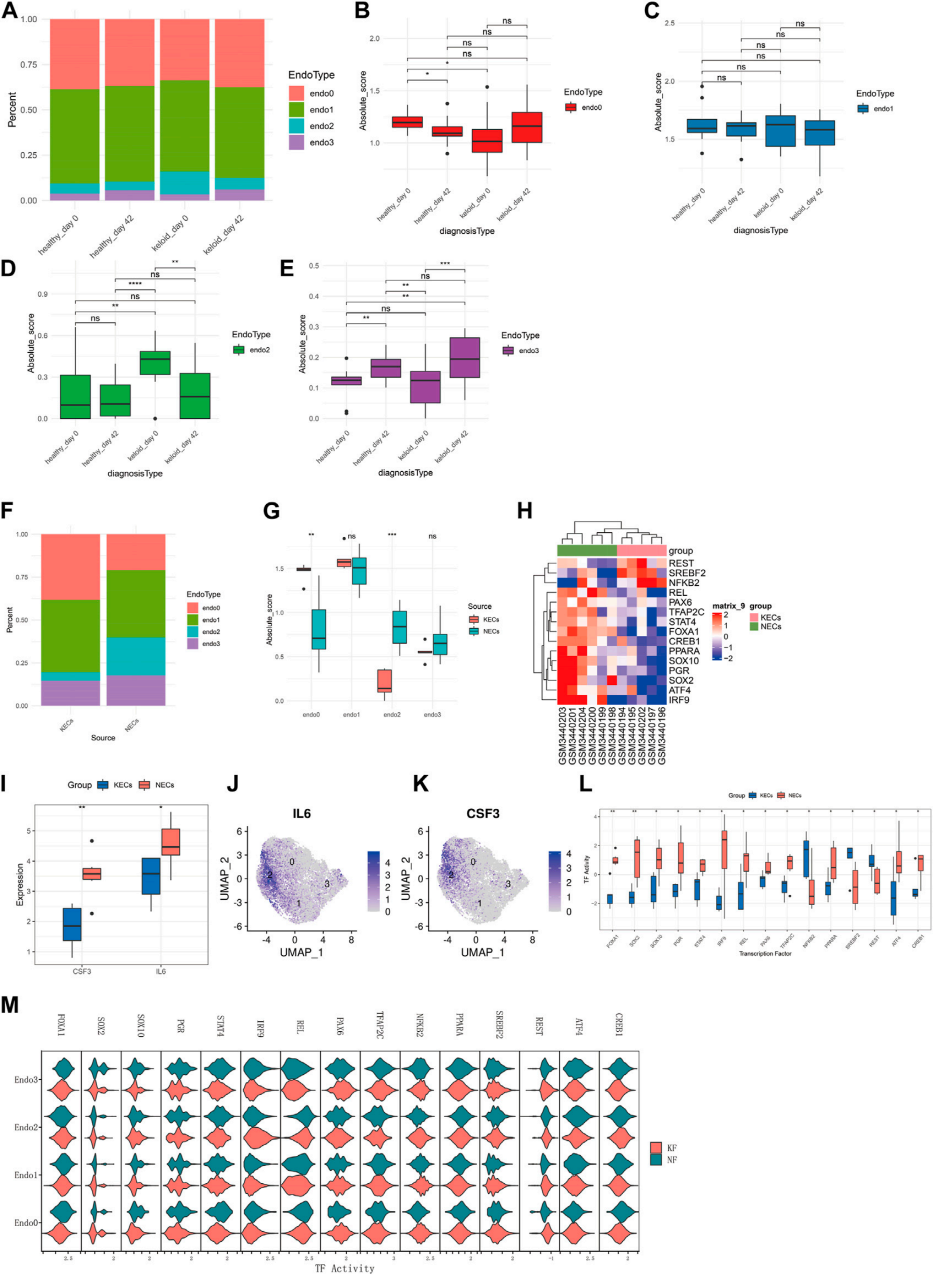
Bulk-RNAseq to explore the relationship between four endothelial cell types and keloidogenesis and development. **(A)** Comparison of the distribution of the four endothelial cells of GSE113621 in different pathological states. **(B–E)** Comparison of the differences between the four endothelial cells respectively. Endo0 scored the least in keloid_day0; Endo1 did not differ significantly between the four groups; Endo2 scored the highest in keloid_day0; Endo3 rose significantly after keloid formation on day 42, both in healthy individuals with normal scars and keloid-prone patients. **(F)** Distribution of the four endothelial cell types (NECs vs. KECs) for the 11 endothelial cell transcriptome data from GSE121618. **(G)** Endo0 was significantly higher in KECs, whereas Endo2 was significantly lower. No significant differences were found for Endo1 and Endo3 (*t*-test). **(H)** Analysis of differential transcription factor activation. **(I)** Comparison of transcription factor activities. **(J)** CSF3 and IL6 were significantly reduced in KECs, which is consistent with the reduced levels of Endo2. **(K,L)** IL6 and CSF3 are significantly enriched in Endo2 at the single-cell level. **(M)** Comparison of the differences in activity of 15 transcription factors in NF and KF at the single-cell level. This result demonstrates that the differences in transcription factor activity between Endo2 and Endo0 are consistent at the single-cell level and at the transcriptome level.

Although at the single cell level we observed that Endo2 to Endo0 differentiation is a major feature of keloid formation, however more evidence is needed to demonstrate this phenomenon as a feature of keloid formation. We deconvoluted transcriptomic data from 64 skin samples of GSE113621 by the CIBERSORTX algorithm. We compared the distribution of four types of endothelial cells in four sources of skin tissue (Figure 3A) and found that Endo0 scores were significantly lower in the skin of the keloid-susceptible population than in the skin of the healthy population. In addition, Endo0 was significantly lower in the skin of the healthy population after scar repair (Figure 3B, *p* < 0.01). Changes in Endo1 were not significantly different in any of the four subgroups (Figure 3C, *p* < 0.01). The proportion of Endo2 was significantly higher in the skin of the keloid-prone population than in the skin of the healthy population. Endo2 was also significantly reduced after keloid formation (Figure 3D, *p* < 0.01). Endo3 was significantly increased in the skin after keloid formation (Figure 3E, *p* < 0.01). Although the results obtained from this dataset are as expected, this may interfere with the analysis of endothelial cells as the samples in this dataset are from intact skin samples. We compared transcriptomic data from 11 endothelial cells in the GSE121618 dataset. The data were mainly derived from endothelial cells from keloids (KECs) and surrounding normal skin (NECs) (Supplementary Figures S1A, S1B). By deconvolution, we found that the proportion of Endo2 was significantly higher in KECs than in NECs, while Endo0 was significantly lower than in NECs. By comparison of their Absolute Scores, we found that Endo0 scored significantly lower in NECs than in KECs (*p* < 0.01), while the opposite was true for Endo2 (*p* < 0.001). Endo1 and Endo3 were not significantly different between the two groups (Figures 3F, G). Transcription factors were analyzed by Dorothea for both groups of samples. The results revealed that REST, SREBF2, and NFΚB2 were significantly activated in KECs, while REL, PAX6, TFAP2C, STAT4, FOXA1, CREB1, PPARA, SOX10, PGR, SOX2, ATF4, and IRF9 were significantly repressed (*p* < 0.05, Figures 3H, I). Comparison of marker genes for Endo2 (IL6, CSF3) showed significantly higher expression of these two genes in NECs than in KECs (*p* < 0.05, Figures 3J–L). This result demonstrates the higher proportion of Endo2 in NECs. Next, we compared these 15 transcription factors at the single cell level between para-keloid (NF) and keloid (KF). The results showed that FOXA1, IRF9, and NFΚB2 were significantly upregulated and REL was significantly down-regulated in Endo2 relative to KF, while FOXA1, SOX10, STAT4, IRF9, and CREB1 were significantly up-regulated and PAX6, NFΚB2, REST were significantly down-regulated in Endo0 (Figure 3M). This result clarifies the process of transcription factor changes in endothelial cells during keloidogenesis.

### Activation of the NFKB signaling pathway is a major hallmark of Endo2

**FIGURE 4:**
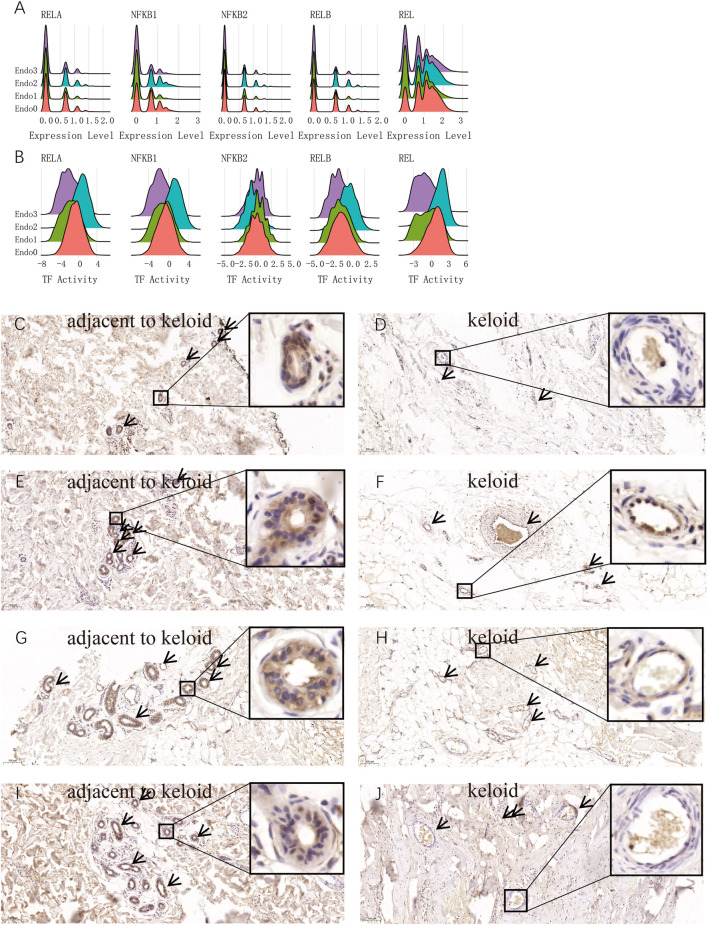
Activation of the NFKB signaling pathway is a major hallmark of activated endothelial cells Endo2. **(A)** Comparison of the distribution of gene expression of the main five transcription factors of NFKB in four endothelial cell types. No significant differences in NFKB signaling pathway-related genes among the four endothelial cells. **(B)** Comparison of the distribution of the five main transcription factor activities of NFKB in the four endothelial cells. The figure shows that RELA, NFKB1, RELB, and REL transcription factors are activated in Endo2, representing the activation of the classical NFKB signaling pathway in Endo2. **(C–J)** RELA immunohistochemical staining of keloid tissues and para-keloid tissues from four keloid patients. Figure C, E, G, I are normal skin tissues adjacent to the keloid; Figure D, F, H, J are lesions of the keloid. NFKB-activated Endo2 was clearly observed in the perivascular distribution and aggregation state.

To further characterize the activation of the NFΚB signaling pathway as a key feature of IL6+CSF3+ Endo2 endothelial cells, we compared the gene expression levels and transcription factor activity of NFΚB1, NFΚB2, RELA, and REL related genes in endothelial cells at the single cell level. The results showed that the expression levels of RELA, NFΚB1, NFΚB2, RELB, and REL were not significantly different in the 3 cell types (Figure 4A), but RELA, NFΚB1, RELB, and REL transcripts were significantly activated by Endo2 and significantly repressed by NFΚB2 (Figure 4B). This result illustrates that activation of the NFΚB signaling pathway is not activated by transcription factors secreted by the vascular endothelium itself, but by transcription factors secreted by perivascular endothelial cells. To further explore the origin of NFΚB-related transcription factors, we analyzed transcription factors at the single cell level. The transcriptome results indicated that the genes for NFΚB1, NFΚB2, and REL were mainly expressed in immune cells. The assessment of transcription factors showed that RELA, NFΚB1, RELB, and REL were activated in immune cells, Endo0 and Endo1 (Supplementary Figures S2A−C). We next performed immunohistochemical staining for RELA on pathological sections of four keloids. The results showed that Endo2 was widely distributed in the para-keloid, whereas in the pathological sites, Endo2 was sparsely distributed in skin tissues showing significant fibrosis (Figures 4C–J). As Endo2 showed aggregated features in the para-keloid tissue, and aggregated apoptotic vascular features in the lesioned tissue. Therefore, we suggest that Endo0 on pathological sections is likely to be the result of loss of vascular features following apoptotic death of Endo2.

### Vascular lesions of inflammatory vessels (Endo2) are typical of keloid

**FIGURE 5:**
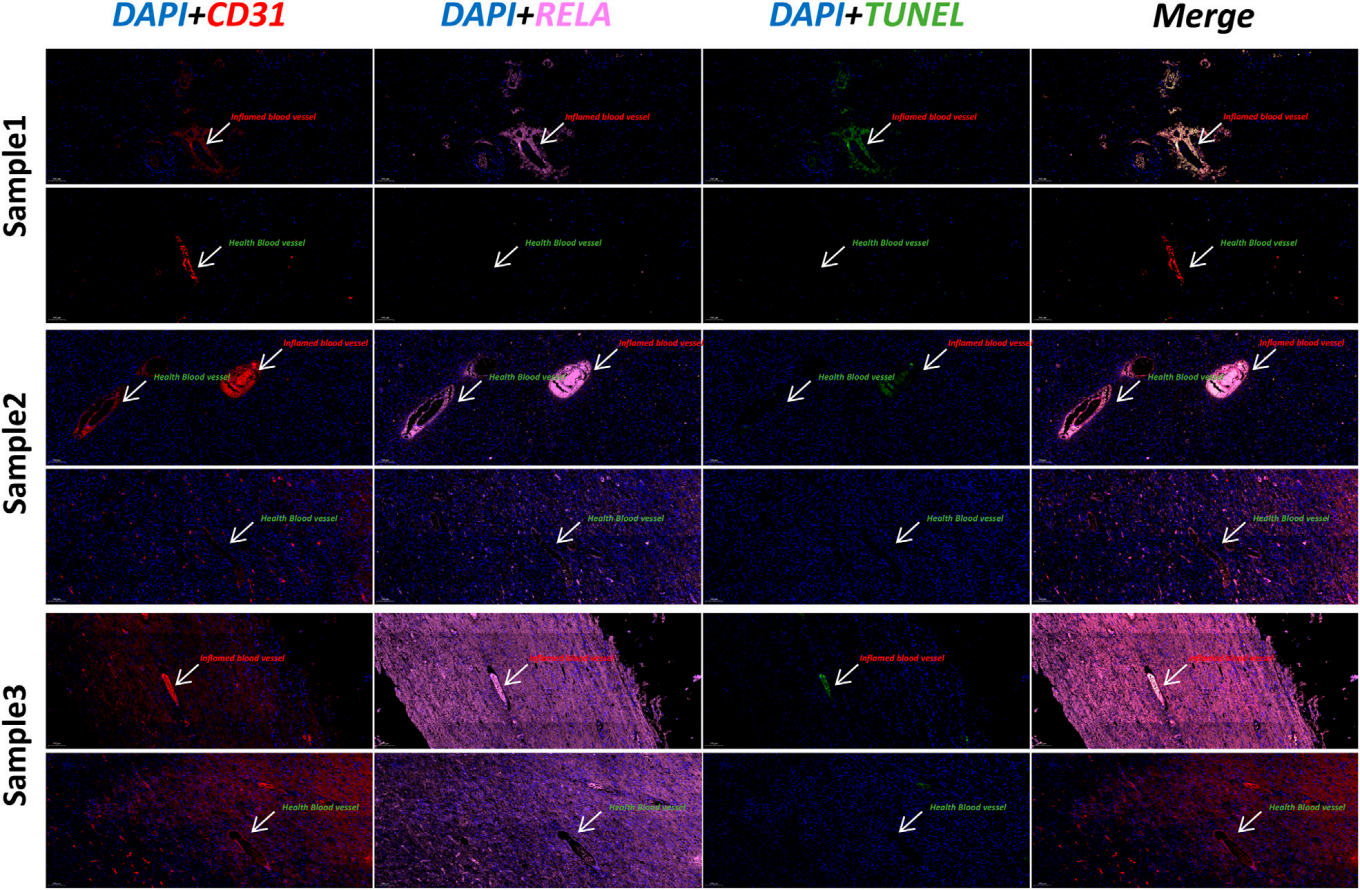
Immunofluorescence staining of keloid and para-keloid (DAPI/CD31/RELA/TUNEL). We used three separate samples of skin tissue for immunofluorescence experiments. We found that the TUNEL signal was stronger in vessels with high RELA expression (Inflamed blood vessels). This result indicates that significant vascular lesions have occurred in RELA-high-expressing Endo2.

We explored keloid lesion tissue and parakeratotic tissue using immunofluorescence techniques. DAPI was used to label nuclei (blue), CD31 to label vascular endothelial cells (red), RELA to label Endo2 vascular endothelial cells (pink), and TUNEL to label apoptotic cells (TUNEL). In all three samples, we observed higher TUNEL fluorescence intensity in inflamed vessels (CD31+RELA+, Endo2) (Figure 5). Normal vessels had low RELA expression and lower TUNEL fluorescence intensity. This result indicates that the inflammatory vessel (Endo2) is already in a vasculopathic state in Keloid. Considering that Endo2 is more abundant in para-keloid, this lesion state may be an early characteristic of Keloid pathogenesis.

## Discussion

Keloid scarring is a condition caused by excessive fibrosis of the skin (Lim et al., 2019). Although keloid scarring has been extensively studied, the key mechanisms leading to keloid formation remain unclear. Numerous studies have shown that keloidogenesis and development are primarily associated with the proliferation of specific fibroblasts (Bettinger et al., 1996; Simman et al., 2003; Xie et al., 2021b). However, endothelial cells also play an important role in the pathogenesis of keloids (Le et al., 2004). The significance of endothelial cell heterogeneity in the formation of keloids is still not well studied. Here, we used single cell sequencing data combined with bulk-RNAseq to explore the major regulatory processes of different types of endothelial cells during keloid formation. These findings will help us to gain insight into the pathogenesis of keloid and provide potential therapeutic targets for clinical treatment of the disease.

Endothelial cells have a markedly heterogeneous variation in the development and progression of keloids [(Limandjaja et al., 2020); (Zhang et al., 2006)]. We identified four subpopulations of endothelial cells in keloid and normal scar tissue by scRNA-seq data: MMP1+ Endo0, FOS + JUN + Endo1, IL6+CSF3+ Endo2, CXCL12 Endo3. The proportion of cells in Endo2 was significantly increased compared to para-keloid tissue, whereas Endo0 was significantly decreased. We obtained the same conclusion in transcriptomic data from endothelial cell bulk-RNAseq. The results of functional enrichment indicated significant activation of the HIF-1 signaling pathway in Endo0. Considering that Endo0 is the terminal differentiation state of endothelial cells, Endo0 is a loss of function of endothelial cells in the diseased tissue (Liu et al., 2004). Due to the significant increase in FOS, JUN expression in Endo1 and the constructed endothelial cell differentiation trajectory (Figures 2E,F), we consider these endothelial cells to be endothelial cells in a resting state (Shih et al., 2010). Endo2 is mainly associated with the activation of signaling pathways such as IL-17 signaling pathway, Fluid shear stress and atherosclerosis, Rheumatoid arthritis, Malaria, Influenza A. In addition, transcription factors of the NFΚB signaling pathway (NFΚB1, RELA, REL, and RELB) were significantly activated in Endo2. These results suggest that Endo2 is a mature, functional vascular endothelial cell (Shih et al., 2010) (Figures 2E,F). Endo3 is mainly associated with Endocrine resistance, Estrogen signaling pathway, Proteoglycans in cancer, Relaxin signaling pathway. Endo3 is also the least differentiated endothelial cell population (Figures 2E,F). Activation of endothelial cells is usually induced by pro-inflammatory cytokines such as TNF-α and IL6, and can promote leukocyte recruitment and attachment to the vessel wall (Ghazizadeh, 2007; Jin et al., 2021). We found that Endo2 is activated by the TNF-α signaling pathway and that it overexpresses IL6 and CSF-3, suggesting that Endo2 is an activated endothelial cell. In addition, we explored the relationship of cellular communication in the tissue microenvironment of keloids *via* Celltalk (Ogawa et al., 2012). The results showed that Endo0, Endo1, and Endo2 interacted with immune cells with a high intensity of CXCL receptor ligands (CXCL2-ACKR1, CXCL8-ACKR1, and CXCL3-ACKR1 etc., Supplementary Figures S2F, S2K). TNF receptor ligand interactions are mainly mediated by immune cells (TNF-TNFRSF1A, TNF-TNFRSF1B, Supplementary Figures S2H, S2M). IL6 receptor ligand interactions are mainly mediated by Endo2 (IL6-IL6R + IL6ST, Supplementary Figures S2G, S2L). The CSF signaling pathway is mainly mediated by immune cells (CSF1-CSF1R, IL34-CSF1R, Supplementary Figures S2J, S2O). Significant activation of the Fluid shear stress and atherosclerosis signaling pathway in Endo2. A large body of evidence suggests that the key risk factor for the formation and progression of scars is mechanical force (Huang et al., 2014). Keloid scars tend to occur in specific body areas, namely the anterior chest, shoulders, scapulae and lower abdomen-suprapubic region. These body parts are characterized by intense and repeated stretching of the skin (Shih and Ardeshir, 2012; Huang et al., 2014). Our results suggest that these sites of repeated stretching are prone to the formation of proliferation of activated endothelial cells (Endo2). It may be the cause of keloids occurring in these areas. In addition, we found that HLA-DRB5 is likewise a highly expressed gene in Endo2 (Figure 1C). In addition to the evidence for a significant association of HLA-DRB5 with the pathogenesis of the version and the major ones, significant associations with keloids have been found by several HLA genes (Brown et al., 2008), in particular HLA-DRB1*15, an association between the HLA and keloids has been observed in both Chinese and Caucasian ethnic groups (Nirodi et al., 2000). The proliferation of activated endothelial cells may therefore be an important mechanism for the formation of keloids. We found in the GSE113621 dataset that Endo2 scores were significantly lower in normal human skin than in keloid-susceptible skin, and similar to normal human skin after keloid formation (Figure 3D). Thus, we can conclude that a high proportion of Endo2 is an important cause of keloid formation and is a typical cutaneous vascular feature of keloid-prone people. Hypoxia due to endothelial hypofunction and microvascular occlusion during keloidogenesis is an important factor in initiating keloid scar maturation. The strong activation of the NFΚB signaling pathway in Endo2 cells suggests that immune cells attack Endo2 by secreting inflammatory factors that cause vascular endothelial cell injury, leading to significant morphological changes in vascular endothelial cells and differentiation of Endo2 into Endo0. Endo0 is the terminal differentiation state of endothelial cells and also represents the apoptotic state in which the vascular endothelium is in following an attack by inflammatory factors. Previously, we found that the markers of Endo2 activation by transcription factor activity analysis are the transcription factors of the NFΚB signaling pathway, of which REL, RELA and NFΚB1 can be used as markers of Endo2. REL, RELA, and NFΚB1 can form the NFΚB complex, which activates the classical NFΚB signaling pathway and causes an inflammatory response (Hristov and Weber, 2004). We explored the differences in RELA in keloid tissues *via* immunohistochemistry. The results demonstrated that in the normal skin surrounding the keloid RELA was expressed in Endo2 and showed aggregated vascular clusters. In contrast, in diseased keloids, RELA was mainly expressed in Endo0, showing a distinct apoptotic profile, and its signal intensity was significantly lower than that of Endo2. The results labeled Endo2 and Endo0 by RELA proteins in the NFΚB signaling pathway and demonstrated pathological differences between the two endothelial cells. We found that Endo3 was in the early state of vascular endothelial cells by CELLTRACE (Figure 2E), and in addition the endothelial progenitor cell markers CD34 and KDR were highly expressed in Endo3 (Yoder, 2012) (Supplementary Figures S3). Thereby, we can assume that Endo3- > Endo1- > Endo2- > and Endo0 represents the entire process of progressive endothelial cell death from the resting state, the activated state and finally apoptosis of endothelial progenitor cells in keloids. Clinically, it has been found that the initial manifestation of keloid occurs when the skin becomes red and changes colour from light red to dark red to purplish red, with the scar colour gradually fading as the scar tissue matures. The change in colour of the scar surface reflects the dynamic process of vascular change within the tissue. This process is consistent with our hypothesis that vascular endothelial cells are driven by inflammatory factors and undergo apoptosis.

In summary, we have systematically analysed the heterogeneity of endothelial cells in keloids with scRNA-seq combined with bulk-RNAseq data to find the pathological process of endothelial cells during keloidogenesis and development. Furthermore, we found that differentiation of IL6+CSF3+ activated endothelial cells to MMP1+ endothelial cells activated by the NFΚB signaling pathway is a major feature of keloid vascular lesions. These findings will help to gain insight into the pathogenesis of keloid and to develop new targeted drugs for vascular endothelial cell lesions.

## Data Availability

The datasets presented in this study can be found in online repositories. The names of the repository/repositories and accession number(s) can be found in the article/Supplementary Material.

## Supplementary material

SUPPLEMENTARY FIGURE S1 (A) Volcano map of differentially expressed KECs and NECs in the GSE121618 dataset. **(B)** Heat map of differentially expressed genes.

SUPPLEMENTARY FIGURE (S2) A Analysis and annotation of subpopulations of endothelial cells and fibroblasts in the single-cell sequencing dataset GSE163973. **(B)** Expression of RELA, NFKB1, NFKB2, RELB, and REL genes in different cells. The expression of RELA, NFKB1, NFKB2, RELAB, and REL was found to be higher in immune cells, Endo0, than in other cells. **(C)** Transcription factor activity of RELA, NFKB1, NFKB2, RELB, and REL in different cell types. The graph shows significant activation of RELA, NFKB1, RELB, and REL in immune cells, Endo2, Endo0, and NFKB2 in Lymphatic endothelial cells. **(D)** Distribution of the three fibroblast subpopulations in the six samples. **(E)** Fibroblast_1 is the typical mesenchymal type of fibroblast and is the main fibroblast type responsible for keloid lesions. **(F,K)** CXCL signaling pathway and types of major interactions. **(G,L)** IL6 signaling pathway network and main interaction types. IL6+ CSF3+ Endo2 is the main player, acting on immune cells and Lymphocyte endothelial **(H, M)** TNF signaling pathway network and main interaction types. Immune cells act mainly through the TNF signaling pathway on other cell types. **(I, N)** CCL signaling pathway network and main types of interaction. Endothelial cells and muscle fibroblast are the main players **(J,O)** CSF signaling pathway network and main interaction types.

SUPPLEMENTARY FIGURE S3 Endo3 is an endothelial progenitor cell. **(A)** CD34 is most highly expressed in Endo3, and **(B)** KDR is more highly expressed in Endo3. These two genes are the main markers of endothelial progenitor cells.
