# Corrigendum: Activation of the NFκB signaling pathway in IL6+CSF3+ vascular endothelial cells promotes the formation of keloids

**DOI:** 10.3389/fbioe.2022.1045496

**Published:** 2022-11-02

**Authors:** Delin Liu, Yidi Zhang, Lisha Zhen, Rong Xu, Zhenling Ji, Zheng Ye

**Affiliations:** ^1^ Department of General Surgery, Institute for Minimally Invasive Surgery, Affiliated Zhongda Hospital, Medical School, Southeast University, Nanjing, China; ^2^ Department of Endocrinology, Affiliated Zhongda Hospital, Medical School, Southeast University, Nanjing, China; ^3^ School of Statistics, Renmin University of China, Beijing, China; ^4^ Beijing Sankuai Online Technology Co., Ltd., Dhaka, Bangladesh

**Keywords:** keloid, IL6, endothelial cells (ECs), scRNAseq, NFkB (RelA)

In the published article, there was an error in affiliations **[1, 2]**. Instead of

“^1^Department of General Surgery, Institute for Minimally Invasive Surgery, Affiliated ZhongDa Hospital, Medical School, Southeast University, Dhaka, Bangladesh

^2^Department of Endcrinology, Affiliated ZhongDa Hospital, Medical School, Southeast University, Dhaka, Bangladesh, it should be

“^1^Department of General Surgery, Institute for Minimally Invasive Surgery, Affiliated ZhongDa Hospital, Medical School, Southeast University, Nanjing, China

^2^Department of Endcrinology, Affiliated ZhongDa Hospital, Medical School, Southeast University, Nanjing, China”

In the published article, there was an error regarding the affiliation(s) for **Delin Liu**. As well as having affiliation “1”, they should also have “Department of Endcrinology, Affiliated Zhongda Hospital, Medical School, Southeast University, Nanjing, China”.

A correction has been made to **Results**, **The endothelial cells of keloid are divided into four subgroups**, [176–180]. This sentence previously stated:

“genes upregulated in Endo1 were mainly involved in Osteoclast differentiation, TNF signaling Endo1 upregulated genes were mainly involved in Osteoclast differentiation, TNF signaling pathway, Apoptosis; Endo2 upregulated genes were mainly involved in IL-17 signaling pathway, Fluid shear stress and atherosclerosis, Rheumatoid arthritis; Endo3 upregulated genes were mainly involved in Endo3 upregulated genes are mainly involved in Endocrine resistance, Estrogen signaling pathway, Proteoglycans in cancer and other signaling pathways”

The corrected sentence appears below:

“genes upregulated in Endo1 were mainly involved in Osteoclast differentiation, TNF signaling pathway, Apoptosis; Endo2 upregulated genes were mainly involved in IL-17 signaling pathway, Fluid shear stress and atherosclerosis, Rheumatoid arthritis; Endo3 upregulated genes were mainly involved in Endocrine resistance, Estrogen signaling pathway, Proteoglycans in cancer and other signaling pathways”

The authors apologize for this error and state that this does not change the scientific conclusions of the article in any way. The original article has been updated.

