# Supplementary figures and images for: Activation of the NFκB signaling pathway in IL6+CSF3+ vascular endothelial cells promotes the formation of keloids
Source: Front Bioeng Biotechnol. 2022 Aug 23;10:917726. doi: 10.3389/fbioe.2022.917726 (PMC9445273; doi:10.3389/fbioe.2022.917726)

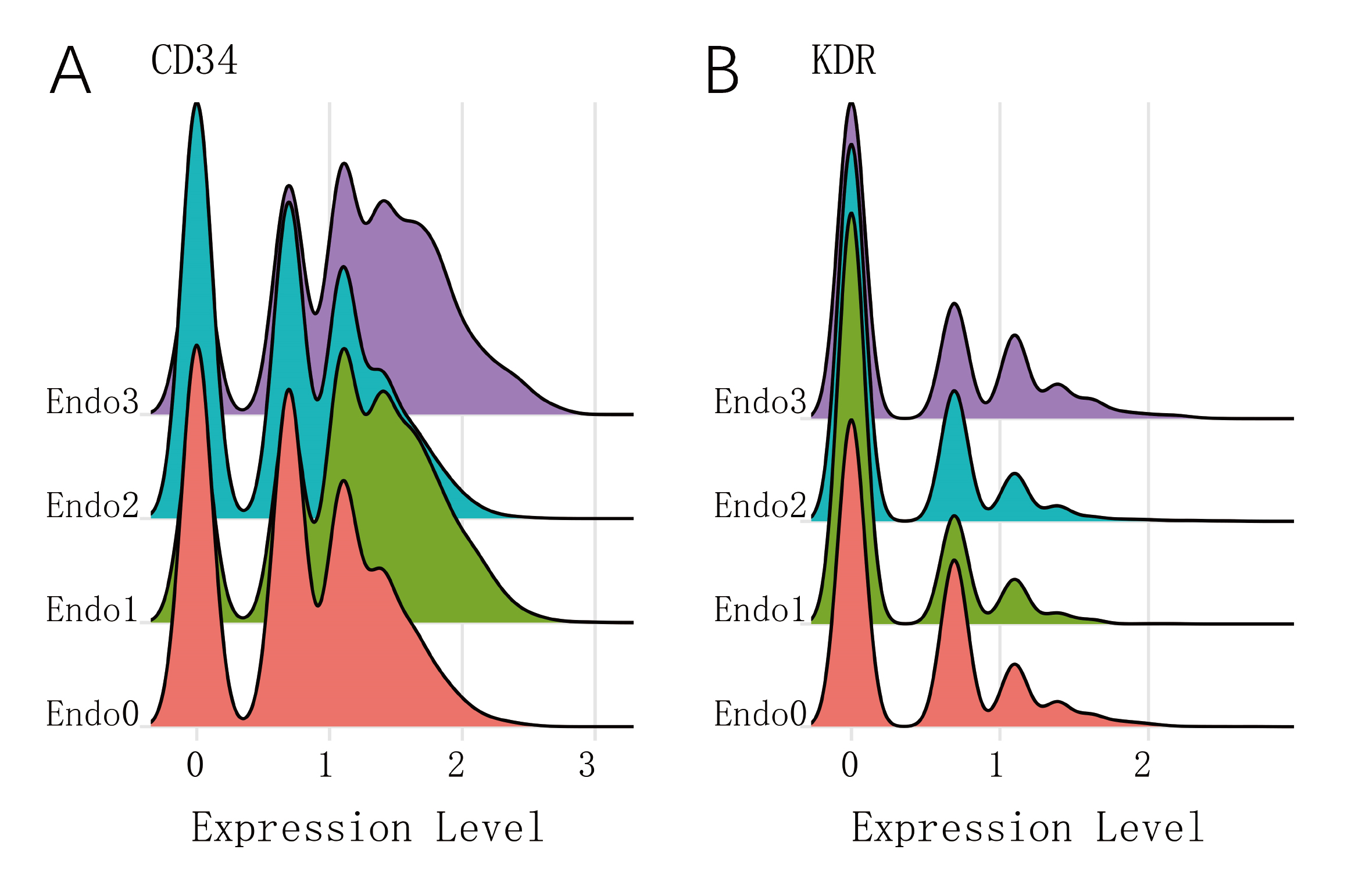

Supplement: Supplementary file 1 [file Image3.JPEG]

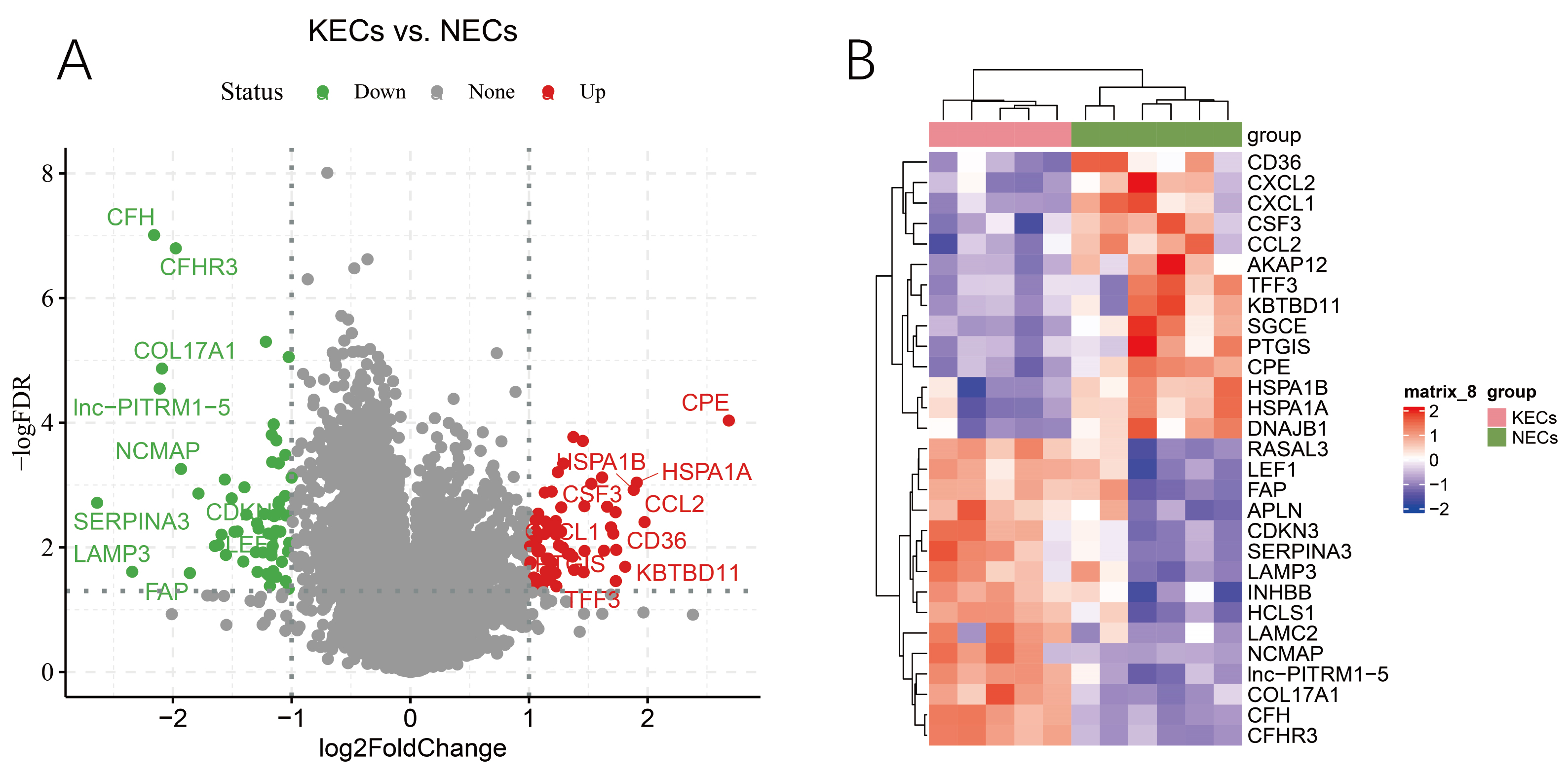

Supplement: Supplementary file 2 [file Image1.JPEG]

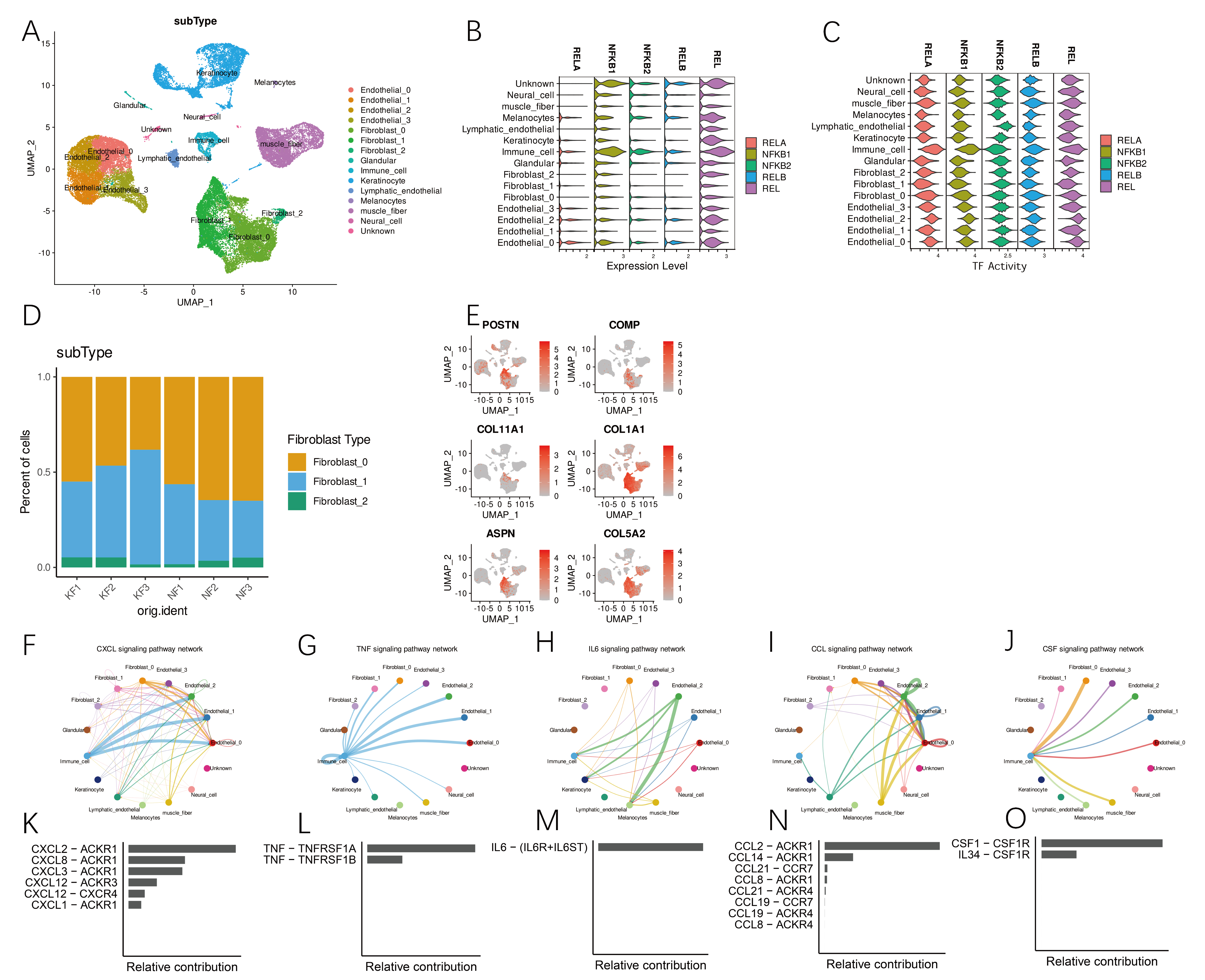

Supplement: Supplementary file 3 [file Image2.JPEG]
